# The Performance Analysis of a Real-Time Integrated INS/GPS Vehicle Navigation System with Abnormal GPS Measurement Elimination

**DOI:** 10.3390/s130810599

**Published:** 2013-08-15

**Authors:** Kai-Wei Chiang, Thanh Trung Duong, Jhen-Kai Liao

**Affiliations:** Department of Geomatics, National Cheng Kung University, 1 University Road, Tainan 701, Taiwan; E-Mails: kwchiang@mail.ncku.edu.tw (K.-W.C.); cacalut1690@gmail.com (J.-K.L.)

**Keywords:** INS, GPS, integration, abnormal measurement

## Abstract

The integration of an Inertial Navigation System (INS) and the Global Positioning System (GPS) is common in mobile mapping and navigation applications to seamlessly determine the position, velocity, and orientation of the mobile platform. In most INS/GPS integrated architectures, the GPS is considered to be an accurate reference with which to correct for the systematic errors of the inertial sensors, which are composed of biases, scale factors and drift. However, the GPS receiver may produce abnormal pseudo-range errors mainly caused by ionospheric delay, tropospheric delay and the multipath effect. These errors degrade the overall position accuracy of an integrated system that uses conventional INS/GPS integration strategies such as loosely coupled (LC) and tightly coupled (TC) schemes. Conventional tightly coupled INS/GPS integration schemes apply the Klobuchar model and the Hopfield model to reduce pseudo-range delays caused by ionospheric delay and tropospheric delay, respectively, but do not address the multipath problem. However, the multipath effect (from reflected GPS signals) affects the position error far more significantly in a consumer-grade GPS receiver than in an expensive, geodetic-grade GPS receiver. To avoid this problem, a new integrated INS/GPS architecture is proposed. The proposed method is described and applied in a real-time integrated system with two integration strategies, namely, loosely coupled and tightly coupled schemes, respectively. To verify the effectiveness of the proposed method, field tests with various scenarios are conducted and the results are compared with a reliable reference system.

## 1. Introduction

The past two decades have seen an increase in the use of positioning and navigation technologies in land vehicle applications. Applications of these technologies in land transportation are numerous, including automated car navigation, emergency assistance, fleet management, person/asset tracking, collision avoidance, environmental monitoring, and automotive assistance. The convergence of location, information management, and communication technologies has created a rapidly emerging market known as location-based services (LBS). LBS is a critical enabling technology that uses location as a filter or magnet to extract relevant information to provide value-added services such as location-aware billing, automated advertising services, and other location-based information sought by the user based on their location. Because of the importance of location information, the market, in turn, has pushed hard for the development of reliable vehicle navigation and guidance systems that provide not only location information but also route guidance and location-sensitive services. Virtually all modern land-vehicle navigation systems integrate two or more complimentary positioning technologies to provide the vehicle's position, velocity, and heading information in a seamless fashion. Typical candidates for these integrated navigation systems are the Global Positioning System (GPS) and Inertial Navigation Systems (INS).

**Figure 1.**
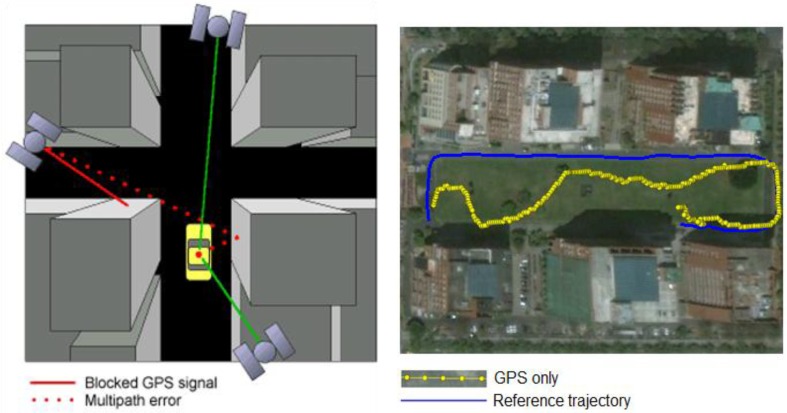
The effect of signal blockage and multipath error in urban areas.

**Figure 2.**
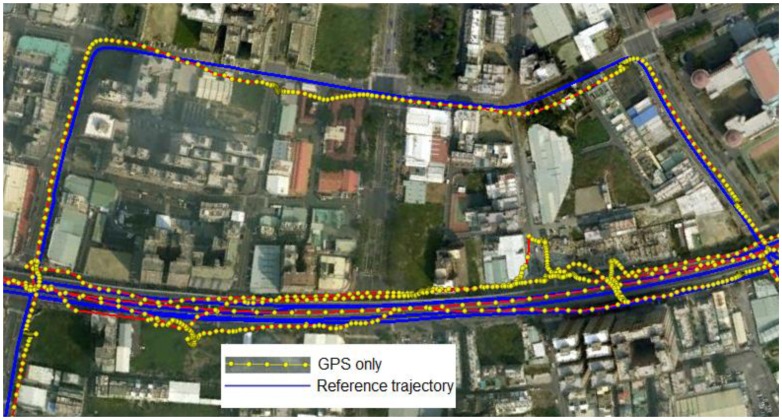
The effect of signal blockage and multipath error in urban areas from a field test.

GPS can provide continuous, accurate positioning given clear lines of sight to more than four satellites. However, the accuracy and the availability of GPS-based vehicle navigation systems are subject to the open-sky conditions and degrade in the presence of signal blockage and reflected signals, as shown in Figure 1 and Figure 2, respectively. The GPS trajectories shown in these figures were obtained from the real-time solutions of a consumer-grade GPS receiver, the AEK 4T from Ublox, and the reference trajectories were obtained from the post-processed, smoothed solutions using a tactical-grade integrated INS/GPS system aided by an odometer, the SPAN-CPT from NovAtel.

When GPS signals are unavailable, the INS can fill in the gaps to provide continuous navigation solutions (position, velocity, and attitude). An Inertial Measurement Unit (IMU) generally contains a set of inertial sensors, e.g., gyroscopes and accelerometers, to provide raw measurements including velocity changes (Δv) and orientation changes (Δθ) in the three directions of its body-fixed coordinate frame. The term “INS” usually refers to an IMU combined with an onboard computer that can provide navigation solutions in the chosen navigation frame directly in real time in addition to compensated raw measurements [1].

Although an integrated navigation system can work in GPS-denied environments, problems include the cost of the inertial sensors and the length of time that the GPS signals are unavailable, which affect its applicability. Tactical-grade or better inertial systems can achieve sufficient position accuracy and sustainability during long-duration GPS signal blockages [1,2]. For example, the high-end, expensive systems can provide less than 3 m real-time position accuracy with a GPS gap lasting one minute. However, the cost of the sophisticated inertial sensors is prohibitive for applications such as the primary navigation module for general land vehicles. For this reason, strap-down Micro-Electro-Mechanical Systems (MEMS) inertial sensors are preferred as the complementary component to GPS for general, seamless vehicle navigation applications. However, the position accuracy of these low-cost inertial sensors degrades rapidly with time when GPS signals are interrupted. The sustainability of an integrated INS/GPS system using currently available commercial MEMS inertial technology in typical GPS-denied environments is thus limited. However, the progress in MEMS inertial sensors has advanced rapidly. Thus, the inclusion of MEMS inertial sensors for general land-vehicle navigation has considerable potential in terms of cost and accuracy [3].

**Figure 3.**
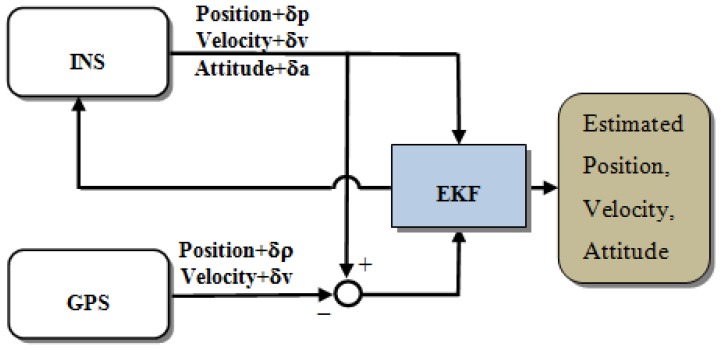
Loosely coupled scheme.

For INS/GPS integration applications, several architectures have been developed [4]. The most common integration scheme used today is the loosely coupled (LC) integration scheme. It is the simplest way of integrating the GPS processing engine into an integrated navigation system. The GPS processing engine calculates position fixes and velocities in the local level frame and then sends the solutions as measurement updates to the main Extended Kalman Filter (EKF). By comparing the navigation solutions provided by the INS mechanization with those provided by the GPS processing engine, the navigation states can be optimally estimated. As shown in Figure 3, the primary advantage of the LC architecture is the simplicity of its implementation; no advanced knowledge of processing GPS measurements is necessary. The disadvantage of this implementation is that the measurement update of the integrated navigation system is possible only when four or more satellites are in view [1,2,4].

**Figure 4.**
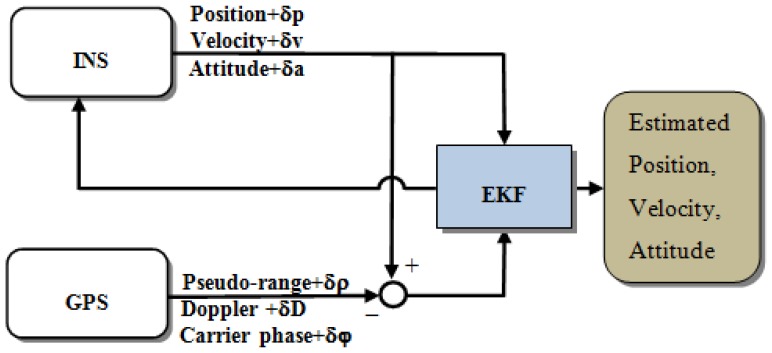
Tightly coupled scheme.

The tightly coupled (TC) integration scheme uses a single EKF to integrate the GPS and IMU measurements. In the TC integration scheme, the GPS pseudo-range, Doppler signal, and carrier-phase measurements are processed directly in the main EKF, as shown in Figure 4. The primary advantage of this architecture is that the raw GPS measurements can be used to update the INS when fewer than four satellites are available. This scheme is particularly beneficial in problematic environments such as downtown city areas, where the reception of GPS satellite signals may be impeded by obstructions.

However, according to Chiang and Huang [5], the EKF implemented with the conventional TC scheme may have serious problems related to the quality of the raw GPS measurements, especially when a low-cost, consumer-grade GPS receiver is used. Most of the expensive, geodetic-grade GPS receivers come with a sophisticated correlator and an antenna designed to manage reflected signals. These expensive GPS receivers usually fail to track a sufficient number of GPS satellites to provide a continuous trajectory when operating in GPS-degraded environments such as urban areas or forests because the reflected signals are actively eliminated [1]. Conversely, the low-cost, consumer-grade GPS receivers tend to provide a continuous although less accurate trajectory because they make use of all the available GPS signals, even the reflected ones that reduce the overall accuracy of the continuous trajectory, as shown in Figure 1 and Figure 2. Conventional, EKF-based TC architectures are sensitive to the quality of the raw GPS measurements. This problem is usually observed in urban and suburban areas because of the reflected GPS signals [4,5].

Real-time INS/GPS systems using MEMS IMUs have been investigated with various computation platforms [6,7]. Liu [8] analyzed the performance of real-time, low-cost MEMS INS/GPS integration with additional constraints in land-based mobile mapping systems (MMSs). In the present study, a land-based, GPS-aided, MEMS inertial navigator is developed using a low-cost, consumer-grade GPS receiver with a modified version of a real-time, TC INS/GPS integration scheme to detect and eliminate abnormal GPS signals caused by multipath and other uncertainties to improve the stability and reliability of the system. In addition, tests using various scenarios are performed to evaluate the performance of the proposed system with actual data.

## 2. Configuration of Proposed Land-Based Global Positioning System Aided Inertial Navigator

The configuration of the proposed land-based, GPS-aided, MEMS inertial navigator and the real-time, modified TC INS/GPS integration scheme are described in detail in this section.

### 2.1. Inertial Navigation System (INS)/Global Positioning System (GPS) Integration

**Figure 5.**
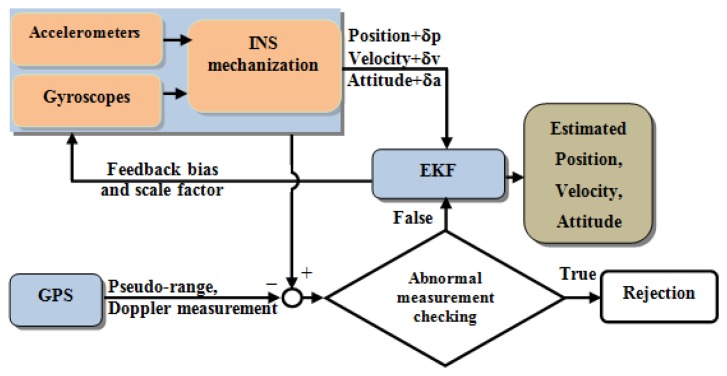
Modified tightly coupled Inertial Navigation System (INS)/Global Positioning System (GPS) integration scheme.

A modified TC INS/GPS integration is proposed in this research as described in the Figure 5. First, the outputs of the IMU, the angular rates sensed by gyroscopes and the specific forces sensed by accelerometers are processed by the INS mechanization to obtain navigation solutions, which are the position, the velocity, and the attitude in the navigation frame. The pseudo-range and Doppler measurements, the raw measurements from the GPS, are pre-processed to eliminate systematic errors such as the satellite clock, tropospheric, and ionospheric errors. Based on the navigation information estimated by the INS mechanization, GPS measurements are then checked to detect and eliminate abnormal measurements before the data-fusion engine is used. This process will be discussed in detail in Section 3. In addition, as a design of a closed-loop, the biases and the scale factors of the accelerometers and the gyroscopes are included in the state vector. Thus, during estimation, these errors are estimated to compensate the raw measurements coming from the IMU. The EKF is used as an estimator for data fusion to obtain estimated navigation solutions.

### 2.2. Additional Aiding Sources

In the presence of GPS outages, additional aids are essential to augment the system to maintain the stability and the accuracy of the navigation solutions. As illustrated in [8], the use of a Zero Velocity Update and a Zero Integrated Heading Rate (ZUPT/ZIHR) can significantly improve the accuracy of the navigation solutions during GPS signal blockages. ZUPT means the occasion stop of the system for short duration for estimating errors of the system and thus bounding the growth of inertial sensor errors. If vehicle stops, the velocity outputs in any directions should be zero. On the other hand, the ZIHR utilize the fact that the heading angle of the vehicle is not changed during the vehicle stops. However, the frequent stopping required for implementing ZUPT/ZIHR is not feasible in practice. In this study, only the Non-Holonomic Constraint (NHC) is applied as an aid during GPS signal outages.

**Figure 6.**
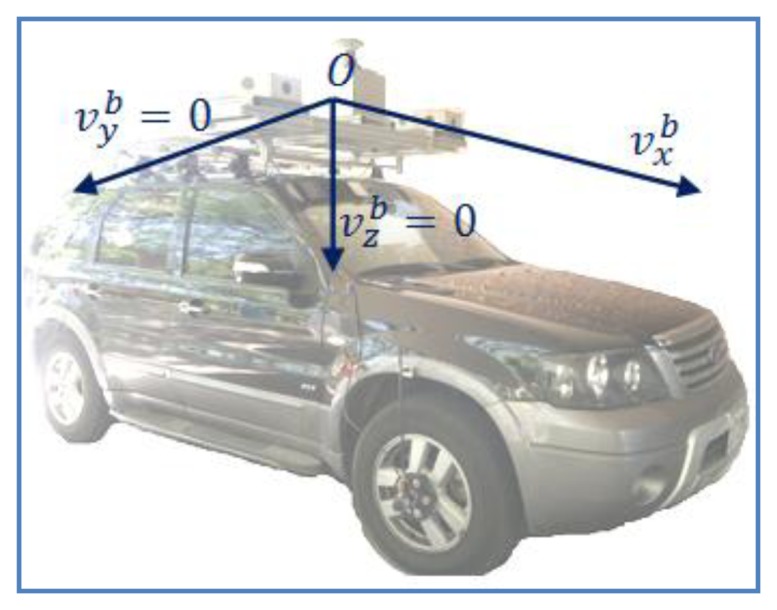
Nonholomonic constraints.

As mentioned in [9], in a land vehicle platform, it is assumed that if the vehicle does not jump off the ground or slide sideways under normal conditions, so the velocities of the vehicle in the plane perpendicular to the forward direction are approximately zero. This assumption becomes a constraint condition for land-based navigation applications. In terms of implementation, the velocity components in the *y* and *z* directions in the body frame will be zero, as given in Equation (1) and illustrated in Figure 6:

(1) $$\{\begin{array}{c} v_{y}^{b}=0\\ v_{z}^{b}=0 \end{array}$$

where the superscript (*b*) denotes the body frame

To derive the measurement update equation for the KF, the velocity in the navigation frame is converted into the body frame:

(2) $$v^{b}={\left(C_{b}^{n}\right)}^{T}v^{n}$$

where superscript (*n*) denotes the navigation frame and *δv^n^* is the rotation matrix from the navigation frame to the body frame.

For the EKF, in which the system model is derived from the error state model, the corresponding error equation to Equation (2) is derived as follows:

(3) $$\delta v^{b}≅C_{n}^{b}\delta v^{n}-C_{n}^{b}(v^{n}\times ){ɛ}^{n}$$

where *δv^b^* is the velocity error vector in the body frame, *δv^n^* is the velocity error vector in the navigation frame, (*v^n^* ×) is the cross-product form of the velocity vector in the navigation frame, and *ε^n^* is the attitude error vector.

The measurement equation can be constructed as follows:

(4) $$z=\left[\begin{array}{c} \delta v_{y}^{b}\\ \delta v_{z}^{b} \end{array}\right]=\left[\begin{array}{ccc} 0 & 1 & 0\\ 0 & 0 & 1 \end{array}\right]C_{n}^{b}\delta v^{n}+\left[\begin{array}{c} {ɛ}_{vy}\\ {ɛ}_{vz} \end{array}\right]$$

where *ε_vy_* and *ε_vz_* are velocity noise in the *y* and *z* directions, respectively.

The NHC is an analytic correction; no additional sensor is required; therefore, it can be applied to any land-based integrated navigation system to improve the navigation accuracy. However, if the assumption of the vehicle behavior is violated, the NHC may cause more noise to the system. Normally, under an open sky, the GPS is more reliable than the NHC. Therefore, in the proposed system, the NHC is activated only when GPS signal outages take place. In addition, the update interval of the NHC is subject to change depending on the quality of the IMU: the higher the IMU quality, the longer the update interval of the NHC should be.

## 3. Model Design for Estimation

Models of the system and the measurements are required for data fusion using EKF [2]. With a high sampling rate and a seamless output, the INS is used to form the system model. GPS measurements and other external aids are used to build the measurement models.

### 3.1. System Model

The system model is derived based on the error model of a strap-down INS in a navigation frame. The “PSI” model is chosen for the integrated navigation system because of its simple attitude error dynamic equation. Details of the derivation of the “PSI” model can be found in [2]. The core of the system dynamic model using the PSI angle error model is expressed in time-continuous form as below:

(5) $$\left[\begin{array}{c} \delta {\dot{r}}^{c}\\ \delta {\dot{v}}^{c}\\ \dot{\psi } \end{array}\right]=\left[\begin{array}{ccc} F_{11} & F_{12} & 0\\ F_{21} & F_{22} & F_{23}\\ 0 & 0 & F_{33} \end{array}\right]\;\left[\begin{array}{c} \delta r^{c}\\ \delta v^{c}\\ \psi \end{array}\right]+\left[\begin{array}{cc} 0 & 0\\ C_{b}^{n} & 0\\ 0 & C_{b}^{n} \end{array}\right]\;\left[\begin{array}{c} \delta f^{b}\\ \delta \omega_{ib}^{b} \end{array}\right]$$

where *δṙ^c^*, *δv̇^c^* and *ψ̇* are the time derivative of the position, velocity and attitude error vector in the computer frame (the local level frame at the location erroneously estimated by the INS mechanization), respectively, $C_{b}^{n}$ is rotation matrix from b-frame to the n-frame, *δf^b^* and $\delta \omega_{ib}^{b}$ are the specific force and angular rate error vector:

(6a) $$F_{11}=[-\omega_{en}^{n}\times ];F_{12}=\left[\begin{array}{ccc} 1 & 0 & 0\\ 0 & 1 & 0\\ 0 & 0 & 1 \end{array}\right];F_{22}=[-(\omega_{en}^{c}+2\omega_{ie}^{c})\times ]$$

(6b) $$F_{23}=[f^{b}\times ];F_{33}=[-(\omega_{en}^{c}+\omega_{ie}^{c})];F_{21}=\left[\begin{array}{ccc} -g/r_{e} & 0 & 0\\ 0 & -g/r & 0\\ 0 & 0 & -2g/(r_{e}+h) \end{array}\right]$$

where *f^b^* is the specific force, *g* is the acceleration of gravity, $\omega_{en}^{n}$ is the rotation rate vector of the navigation frame (n-frame) relative to the earth frame, $\omega_{ie}^{c}$ is the rotation rate vector of the earth frame relative to the inertial frame expressed in computer frame, *r_e_* is the earth radius, *h* is the ellipsoid high. Equation (5) can be rewritten as follows:

(7) $${\dot{x}}_{1}=F_{1}x_{1}+G_{1}u_{1}$$

where:

(8) $$x_{1}=\left[\begin{array}{c} \delta r^{c}\\ \delta v^{c}\\ \psi \end{array}\right];F_{1}=\left[\begin{array}{ccc} F_{11} & F_{12} & 0\\ F_{21} & F_{22} & F_{23}\\ 0 & 0 & F_{33} \end{array}\right];G_{1}=\left[\begin{array}{cc} 0 & 0\\ C_{b}^{n} & 0\\ 0 & C_{b}^{n} \end{array}\right];u_{1}=\left[\begin{array}{c} \delta f^{b}\\ \delta \omega_{ib}^{b} \end{array}\right]$$

For the closed-loop architecture, the biases and the scale factors of the gyroscopes and the accelerometers are considered as unknown parameters. These parameters are included in the state vector to be estimated in every computational cycle of the KF. The models for these parameters are as follows:

(9a) $${\dot{b}}_{a}=\text{diag}(c_{ab})b_{a}+w_{ab}$$

(9b) $${\dot{b}}_{g}=\text{diag}(c_{gb})b_{g}+w_{gb}$$

(9c) $${\dot{s}}_{a}=\text{diag}(c_{as})s_{a}+w_{as}$$

(9d) $${\dot{s}}_{g}=\text{diag}(c_{gs})s_{g}+w_{gs}$$

where *b_a_*, *b_g_*, *s_a_* and *s_g_* are biases and scale factors of the accelerometers and the gyroscopes, respectively, *c_ab_*, *c_gb_*, *c_as_*, *c_gs_*, *w_ab_*, *w_gb_*, *w_as_*, and *w_gs_* are the discrete-time sensor error model parameters that can be determined from the first term of Gauss-Markov processes.

Let:

(10a) $$x_{2}=\left[\begin{array}{c} b_{a}\\ b_{g}\\ s_{a}\\ s_{g} \end{array}\right];G_{2}=\left[\begin{array}{cccc} 1 & 0 & 0 & 0\\ 0 & 1 & 0 & 0\\ 0 & 0 & 1 & 0\\ 0 & 0 & 0 & 1 \end{array}\right];u_{2}=\left[\begin{array}{c} w_{ab}\\ w_{gb}\\ w_{as}\\ w_{gs} \end{array}\right]$$

(10b) $$F_{2}=\left[\begin{array}{cccc} \text{diag}(c_{ab}) & 0 & 0 & 0\\ 0 & \text{diag}(c_{ab}) & 0 & 0\\ 0 & 0 & \text{diag}(c_{ab}) & 0\\ 0 & 0 & 0 & \text{diag}(c_{ab}) \end{array}\right]$$

Equation (9) can be rewritten as follows:

(11) $${\dot{x}}_{2}=F_{2}x_{2}+G_{2}u_{2}$$

Combining Equations (7) and (12) yields the following:

(12) $$\left[\begin{array}{c} {\dot{x}}_{1}\\ {\dot{x}}_{2} \end{array}\right]=\left[\begin{array}{cc} F_{1} & 0\\ 0 & F_{2} \end{array}\right]\;\left[\begin{array}{c} x_{1}\\ x_{2} \end{array}\right]+\left[\begin{array}{cc} G_{1} & 0\\ 0 & G_{2} \end{array}\right]\;\left[\begin{array}{c} u_{1}\\ u_{2} \end{array}\right]$$

Equation (13) can be rewritten as follows:

(13) $$\dot{x}=Fx+Gu$$

Equation (14) is transformed into a discrete-time form based on [2]:

(14) $$x_{k}=\Phi_{k-1;k}x_{k-1}+w_{k}$$

where $x_{k}={\left[\delta r\;\delta v\;\delta \psi \;b_{a}\;b_{g}\;s_{a}\;s_{g}\right]}_{21\times 1}^{T}$ is the state vector at time (epoch) *k*, Φ*_k_*_−1;_*_k_* is the state transition matrix from epoch *k* − *1* to *k*, and *w_k_* is the system noise. In KF-based, the system noise is assumed to have normal distribution with zero mean and variance *Q_k_*, *w_k_* ∼ *N*(0, *Q_k_*). In fact, the system noise *w_k_* is not usually correctly estimated but can be modeled by the representation of the system noise model *Q_k_*. In theory, $Q_{k}=E[w_{k}w_{k}^{T}]$, however, because the *w_k_* is not directly estimated at every epoch *k*, the *Q_k_* is normally fixed overtime and is modeled by IMU calibration based on Gauss-Markov processes. The IMU calibration process can be found in [10].

### 3.2. Measurement Model

For the LC scheme, the aiding measurements are position and velocity provided by the GPS receiver. For the EKF, the measurement model is as follows:

(15) $$z=\left[\begin{array}{c} r_{\text{INS}}^{e}-r_{\text{GPS}}^{e}\\ v_{\text{INS}}^{e}-v_{\text{GPS}}^{e} \end{array}\right]=\left[\begin{array}{cc} H_{r} & 0\\ 0 & H_{v} \end{array}\right]\;\left[\begin{array}{c} \delta r^{e}\\ \delta v^{e} \end{array}\right]+\left[\begin{array}{c} {ɛ}_{r}\\ {ɛ}_{v} \end{array}\right]$$

where $H_{r}=H_{v}=\left[\begin{array}{ccc} 1 & 0 & 0\\ 0 & 1 & 0\\ 0 & 0 & 1 \end{array}\right]$ are mapping matrices, *δr^e^* is the position error vector, *δv^e^* is the velocity vector expressed in the earth-centered, earth-fixed frame (ECEF or e-frame), and *ε_r_* and *ε_v_* are the position and velocity noise, respectively. Identical to the system noise, the measurement noises vector *ε* = [*ε_r_, ε_v_*] *^T^* is not directly estimated, but is accounted in the EKF by a representation of the measurement noise model *R*, where *R* is modeled based on the GPS position and velocity uncertainty.

For the TC scheme, the pseudo-range and Doppler measurements are used to aid the INS mechanization. Pseudo-range is the erroneous rage from the GPS receiver to the satellite. The original pseudo-range measurement equation derived by [11] is shown as:

(16) $$\rho =r+c(\delta t_{u}+\delta t_{s})+I_{\rho }+T_{\rho }+{ɛ}_{\rho }$$

where *ρ* is the pseudo-range, *r* is the true range between the receiver and the satellite, *δt_s_* is the satellite clock error, *δt_u_* is the clock synchronization error, *I_ρ_* and *T_ρ_* are the delays associated with the transmission of the signal through the ionosphere and troposphere, respectively, *c* is the speed of light, and *ε_ρ_* is pseudo-range noise.

In this research ionospheric, tropospheric and satellite clock errors are estimated before using pseudo-range in EKF. The Klobuchar model [12] is used to account for ionospheric error and Hofield model [13] is used to eliminate tropospheric error. The satellite clock error can be estimated based on information from ephemeris message. After error correction, the Equation (17) becomes:

(17) $$\rho =r+c\delta t_{u}+{ɛ}_{\rho }$$

Doppler measurement (Doppler shift) is the difference between the frequency of the received signal (carrier phase received at the receiver) and the frequency at the source (carrier phase transmitted at the satellite) [11]. The equation of the Doppler measurement is expressed as:

(18) $$D=f_{r}-f_{s}=\frac{{\dot{r}}_{rs}}{\lambda }+{ɛ}_{D}$$

where *f_r_* is the received frequency from the receiver, *f_s_* is the original frequency transmitted by the satellite, *ṙ_rs_* is the change line of sight distance between the receiver and the satellite, λ is the signal wavelength ( $\lambda_{f_{L1}}=\frac{c}{f_{L1}}\approx 0.19m$, where *c* is the speed of light, f_L1_ is the GPS L1 carrier frequency), *ε_D_* is the Doppler measurement noise.

Account for the GPS receiver clock drift error, the Doppler measurement equation can be rewritten as:

(19) $$D=e^{T}\frac{v_{r}-v_{s}}{\lambda }+\frac{c\delta t_{ru}}{\lambda }+{ɛ}_{D}$$

where *e* is line of sight vector, *v_r_* is the receiver velocity, and *v_s_* is the satellite velocity, *cδt_ru_* is the receiver clock drift error.

From Equations (18) and (20), with *m* satellites observed, the pseudo-range and Doppler measurements can be modeled for EKF as:

(20) $$\left[\begin{array}{c} z_{\rho 1}\\ z_{\rho 2}\\ \cdots \\ z_{\rho m} \end{array}\right]=\left[\begin{array}{c} \rho_{\text{INS},1}^{e}-\rho_{\text{GPS},1}^{e}\\ \rho_{\text{INS},2}^{e}-\rho_{\text{GPS},2}^{e}\\ \cdots \\ \rho_{\text{INS},m}^{e}-\rho_{\text{GPS},m}^{e} \end{array}\right]=\left[\begin{array}{ccc} e_{1x} & e_{1y} & e_{1z}\\ e_{2x} & e_{2y} & e_{2z}\\ \cdots & \cdots & \cdots \\ e_{mx} & e_{my} & e_{mz} \end{array}\right]\delta r^{e}+\left[\begin{array}{c} c\delta t_{u}\\ c\delta t_{u}\\ \cdots \\ c\delta t_{u} \end{array}\right]+\left[\begin{array}{c} {ɛ}_{\rho 1}\\ {ɛ}_{\rho 2}\\ \cdots \\ {ɛ}_{\rho m} \end{array}\right]$$

(21) $$\left[\begin{array}{c} z_{D1}\\ z_{D2}\\ \cdots \\ z_{Dm} \end{array}\right]=\left[\begin{array}{c} D_{\text{INS},1}^{e}-D_{\text{GPS},1}^{e}\\ D_{\text{INS},2}^{e}-D_{\text{GPS},2}^{e}\\ \cdots \\ D_{\text{INS},m}^{e}-D_{\text{GPS},m}^{e} \end{array}\right]=\frac{1}{\lambda }\left[\begin{array}{ccc} e_{1x} & e_{1y} & e_{1z}\\ e_{2x} & e_{2y} & e_{2z}\\ \cdots & \cdots & \cdots \\ e_{mx} & e_{my} & e_{mz} \end{array}\right]\delta v^{e}+\frac{1}{\lambda }\left[\begin{array}{c} c\delta t_{ru}\\ c\delta t_{ru}\\ \cdots \\ c\delta t_{ru} \end{array}\right]+\left[\begin{array}{c} {ɛ}_{D1}\\ {ɛ}_{D2}\\ \cdots \\ {ɛ}_{Dm} \end{array}\right]$$

where the superscript *e* denotes the e-frame, *e_kx_*, *e_ky_* and *e_kz_* are the projected components of the line of sight to satellite *k* in the *x*, *y*, and *z* axes in the e-frame, respectively, $\rho_{\text{INS},k}^{e}$ and $D_{\text{INS},k}^{e}$ are the predictions of the pseudo-range and Doppler measurements by the INS with satellite *k* and determined as:

(22) $$\rho_{\text{INS},k}^{e}=\sqrt{{\left(x_{\text{INS}}^{e}-x_{SV,k}^{e}\right)}^{2}+{\left(y_{\text{INS}}^{e}-y_{SV,k}^{e}\right)}^{2}+{\left(z_{\text{INS}}^{e}-z_{SV,k}^{e}\right)}^{2}}$$

(23) $$D_{\text{INS},k}^{e}=e_{k}^{T}\frac{v_{\text{INS}}^{e}-v_{SV,k}^{e}}{\lambda }$$

From Equations (16), (21) and (22), after some transformations, the measurement model can be expressed in a form required by the EKF:

(24) $$z_{k}=H_{k}x_{k}+{ɛ}_{k}$$

In the TC INS/GPS integration scheme with GPS pseudo-range and Doppler phase measurements, the receiver clock error and the clock drift error are included in the system dynamic model:

(25) $$\left[\begin{array}{c} c\delta {\dot{t}}_{u}\\ c\delta {\dot{t}}_{ru} \end{array}\right]=\left[\begin{array}{cc} 1 & 0\\ 0 & 1 \end{array}\right]\;\left[\begin{array}{c} c\delta t_{u}\\ c\delta t_{ru} \end{array}\right]+\left[\begin{array}{c} w_{tu}\\ w_{\mathrm{tru}} \end{array}\right]$$

where *w_tu_* and *w_tru_* are the white noise of the receiver clock error and the clock drift error.

The state vector becomes:

(26) $$x={\left[\delta r\;\delta v\;\delta \psi \;b_{a}\;b_{g}\;s_{a}\;s_{g}\;c\delta t_{u}\;c\delta t_{ru}\right]}_{23\times 1}^{T}$$

### 3.3. Data Fusion Strategies

This section first describes the use of the EKF to estimate the state variables and their corresponding covariances based on the system and measurement models. Subsequently, a strategy is proposed to eliminate abnormal GPS measurements.

#### 3.3.1. Estimation with EKF

Based on the system model expressed in Equation (15), the states and the associated covariance matrix at time *k* are predicted based on the states and the covariance at time *k* o *1*:

(27) $${\hat{x}}_{k}^{-}=\phi_{k-1;k}{\hat{x}}_{k-1}$$

(28) $$P_{k}^{-}=\phi_{k-1;k}P_{k-1}\phi_{k-1;k}^{T}+Q_{k}$$

Whenever aiding measurements are available, the estimated states and their covariance matrix are updated based on the following equations:

(29) $${\hat{x}}_{k}={\hat{x}}_{k}^{-}+K_{k}(z_{k}-H{\hat{x}}_{k}^{-})$$

(30) $$P_{k}=P_{k}^{-}-K_{k}H_{k}P_{k}^{-}$$

where ${\hat{x}}_{k}^{-}$ and $P_{k}^{-}$ are the predicted states and the associated covariance matrix at time *k*, respectively, *x̂_k_*_−1_ and *P_k_*_−1_ are the estimated states and the associated covariance matrix at time *k* − *1*, respectively, and *x̂_k_* and *P_k_* are the estimated states and the associated covariance at time *k*, respectively, *K_k_* is the Kalman gain matrix, which is determined by the following equation:

(31) $$K_{k}=P_{k}^{-}H_{k}^{T}{\left(H_{k}P_{k}^{-}H_{k}^{T}+R_{k}\right)}^{-1}$$

where $R_{k}=E\{{ɛ}_{k}{ɛ}_{k}^{T}\}$.

Normally, the updated estimates are better than the estimates at the prediction step. Thus, the estimated biases and scale factors in the update steps are used to compensate the IMU measurements in subsequent epochs:

(32) $$\Delta \theta_{k+1}=(\Delta {\hat{\theta }}_{k}-b_{g})/(1+s_{g})$$

(33) $$\Delta v_{k+1}=(\Delta {\hat{v}}_{k}-b_{a})/(1+s_{a})$$

where Δ*θ_k_*_+ 1_ and Δ*v_k_*_+ 1_ are the increments in the angle and the velocity at time *k* + *1*, Δ*θ̂_k_* and Δ*v̂_k_* are the increments in the angle and the velocity estimated at time *k*, respectively.

As discussed in Section 1, the accuracy of the GPS measurements is independent of time but dependent on the operating environment. This means that the GPS measurements may deteriorate at any time in a GPS-degraded environment, such as in an urban canyon or a tunnel. In contrast, the solutions from the INS are continuously provided in any environment and are accurate in the short term. Using this characteristic, the predicted states based on the INS mechanization are used to decide if the GPS measurements are sufficiently reliable to be used to perform measurement update in the EKF. This problem is considered for both the LC and TC schemes. If the position or the pseudo-range is biased because of abnormal signals, then the velocity and Doppler measurement are also biased. Therefore, only the position and the pseudo-range are considered for abnormal GPS measurement rejection in these schemes, respectively. Two methods of abnormal GPS measurement rejection are considered in this research, an analytic method and a statistical method.

#### 3.3.2. Analytic Method for Rejecting Abnormal Global Positioning System Measurements

In the LC scheme, the difference vector that forms the aiding position error measurement vector in Equation (16) can be extended as follows:

(34) $$z_{r}=r_{\text{INS}}-r_{\text{GPS}}$$

In ideal conditions, if no error exists on INS and GPS positions, the norm of the vector *z_r_* is zero. Because Kalman filtering theory assumes that all noises have a Gaussian distribution, the norm of vector *z_r_* also have a normal distribution with zero mean and a standard deviation *σ_z_*, where:

(35) $$\sigma_{z}=\sqrt{\sigma_{rINS}^{2}+\sigma_{r\text{GPS}}^{2}}$$

Based on probability theory, if the norm of vector *z_r_* has normal distribution, it is limited by a value with a certain confidence level:

(36) $$|z_{r}|\leq {ɛ}_{\beta \cdot }\sigma_{z}$$

where *ε_β_* is a critical value for the confidence level *β* (e.g., *ε*_95%_ = 196), |*z_r_*| is the norm of vector *z_r_*, determined by the below equation:

(37a) $$|z_{r}|=\sqrt{z_{rx}^{2}+z_{ry}^{2}+z_{rz}^{2}}$$

Based on the criteria shown in Equation (37), if |*z_r_*| > *ε_β_*. *σ_z_*, then the specified GPS measurement should be rejected.

The variable *σ_rGPS_* shown in Equation (36) is the standard deviation of the GPS position. Its magnitude depends on the quality of the GPS receiver and the positioning technique. With a single-frequency GPS receiver in SPP mode, the nominal value of *σ_rGPS_* varies from 3 to 6 m [11]. The variable *σ_rINS_* shown in Equation (36) is the standard deviation of the position predicted by the INS. Its magnitude depends on the performance of the IMU and the length of time that the INS is in stand-alone mode or the duration of the GPS outage. An empirical equation of *σ_rINS_* is constructed as a function of gyroscope bias, accelerometer bias (the major error sources of an IMU) and time based on [1], which is given below:

(37) $$\sigma_{rINS}=\sqrt{{\left(gb_{g}\frac{t^{3}}{6}\right)}^{2}+{\left(b_{a}\frac{t^{2}}{2}\right)}^{2}}$$

where *g* is the acceleration of gravity, *b_g_* is the gyroscope bias, *b_a_* is the accelerometer bias, which is obtained from a calibration or from the estimates of the previous EKF update with a closed-loop architecture, and *t* is the time of the INS in stand-alone mode, which is measured from the previous GPS update.

For example, an IMU with a gyroscope bias of 20 degrees/h and an accelerometer bias of 10 milli-g, the value of *σ_rINS_* equals 4.5 m for a 10-s GPS outage. With the standard deviation of the nominal GPS position chosen to be 5 m, the value of *σ_z_* is 6.7 m, and the limiting value with a confidence level of 95% is 13.2 m.

In the modified TC mode, the difference between the measured GPS pseudo-range and the pseudo-range estimated by the INS is considered, *i.e.*,:

(38) $$z_{\rho }=\rho_{\text{INS}}-\rho_{\text{GPS}}$$

An error criterion is as follows:

(39) $$\left\|z_{\rho }\right\|\leq {ɛ}_{\beta \cdot }\sigma_{\rho }$$

where ‖*z_ρ_*‖ is the absolute value of *z_ρ_*:

(40) $$\sigma_{\rho }=\sqrt{{\sigma^{2}}_{\rho \text{INS}}+\sigma_{\rho \text{GPS}}^{2}}$$

*σ_ρINS_* can be derived from Equation (23) by the variance propagation law. Ignoring the satellite positional uncertainty, the pseudo-range standard deviation estimated by INS becomes:

(41) $$\sigma_{\rho \text{INS}}^{2}={\left(\frac{x_{\text{INS}}^{e}-x_{SV,k}^{e}}{\rho_{\text{INS}}^{e}}\right)}^{2}\sigma_{\text{xINS}}^{2}+{\left(\frac{y_{\text{INS}}^{e}-y_{SV,k}^{e}}{\rho_{\text{INS}}^{e}}\right)}^{2}\sigma_{\text{yINS}}^{2}+{\left(\frac{z_{\text{INS}}^{e}-z_{SV,k}^{e}}{\rho_{\text{INS}}^{e}}\right)}^{2}\sigma_{\text{zINS}}^{2}$$

Assuming that *σ_xINS_* = *σ_yINS_* = *σ_zINS_* = *σ_rINS_* the Equation (42) becomes:

(42) $$\sigma_{\rho \text{INS}}=\sigma_{rINS}$$

The variable *σ_ρGPS_* is the pseudo-range standard deviation. It can be modeled based on the satellite clock and ephemeris, the atmospheric propagation, and the multipath effect. For a single-frequency GPS receiver, a nominal value of the range error is estimated as follows [11]:

(43) $$\sigma_{\rho \text{GPS}}=\sqrt{\sigma_{RE/CS}^{2}+\sigma_{RE/P}^{2}+\sigma_{RE/RNM}^{2}}\approx 6\text{m}$$

where *σ_RE/CS_* is the control-segment standard deviation, *σ_RE/P_* is the atmospheric propagation standard deviation, and *σ_RE/RNM_* is the uncertainty due to the multipath effect.

#### 3.3.3. Statistical Method for Rejecting Abnormal Global Positioning System Measurements

**Figure 7.**
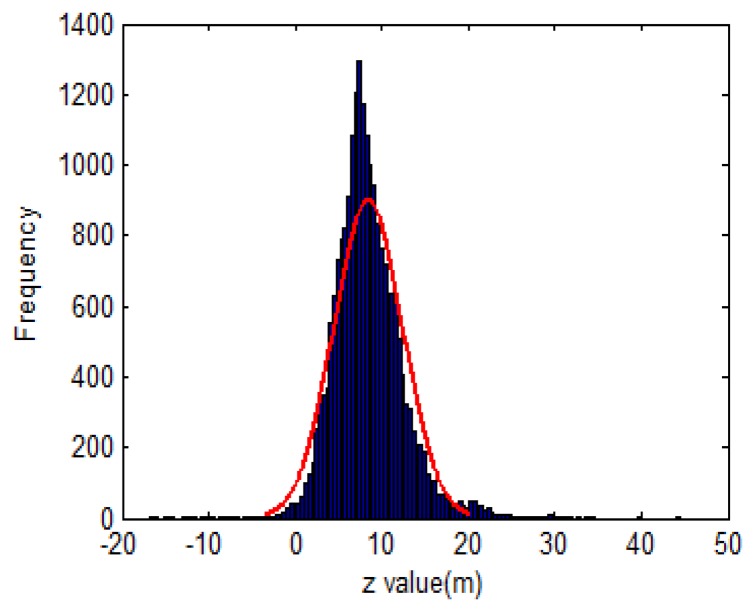
Histogram of *z_ρ_* variable.

One of the assumptions following Kalman filtering theory is that |*z_r_*| in Equation (37) and *z_ρ_* in Equation (40) have normal distributions with zero mean and associated standard deviation *σ_z_* and *σ_ρ_*, respectively. However, the output of the INS contains not only the white noise but also systematic errors such as the biases and scale factors. Therefore, the aforementioned statistical assumption is violated. Figure 7 shows the distribution of *z_ρ_* in a field test. In addition, the uncertainty of the INS and GPS measurements in Equations (38), (41) and (44) are not always correctly estimated because intensive laboratory calibrations are required to determine the systematic errors of the INS and GPS. To overcome these issues, a statistical method using another variable is proposed. From Equations (16) in LC scheme and Equation (21) in the TC scheme, the aiding measurements can be revised as follows:

(44a) $$z_{r}=r_{\text{INS}}^{e}-r_{\text{GPS}}^{e}=H_{r}\delta r^{e}+{ɛ}_{r}$$

(44b) $$z_{\rho }=\rho_{\text{INS}}^{e}-\rho_{\text{GPS}}^{e}=e^{T}\delta r^{e}+c\delta t_{u}+{ɛ}_{\rho }$$

Assuming:

(45a) $$\text{inn}o_{r}=z_{r}-H_{r}\delta r^{e}$$

(46b) $$\text{inn}o_{\rho }=z_{\rho }-(e^{T}\delta r^{e}+c\delta t_{u})$$

**Figure 8.**
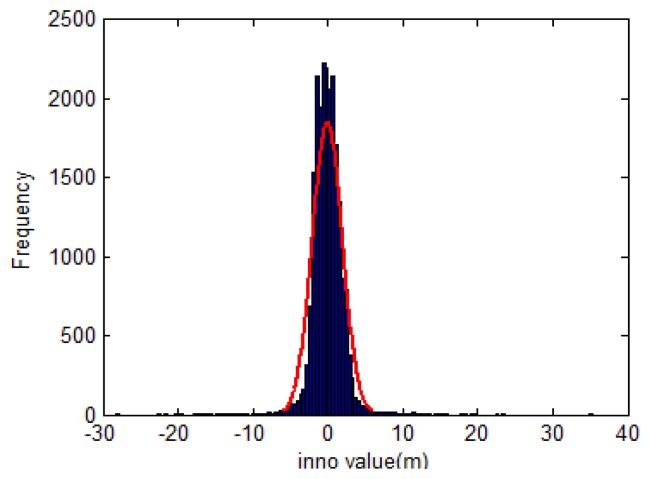
Histogram of *inno* variable.

From Equation (46), if *z_r_* and *σ_ρ_* contain INS systematic errors, then $\delta_{r}^{e}=r_{*}^{e}-r_{\text{INS}}^{r}$ (where $r_{*}^{e}$ is the true position with no error) represents the same situation, and the differences in Equation (46) can eliminate the INS systematic error. This means that generally, the *inno* contains only white noise, and therefore *inno* has a normal distribution with zero mean and a standard deviation *σ_inno_*. Figure 8 shows the histogram of a set of *inno* calculated from a field test data. Since *σ_inno_* is determined, the abnormal GPS measurements are detected and eliminated following the rules in Equation (37) with the LC scheme, and Equation (40) with the TC scheme. In this research, *σ_inno_* is initially determined from the alignment and adaptively updated during operation using following equations:

(46) $$m_{\text{inno}(n)}=m_{\text{inno}(n-1)}+\frac{\text{inn}o_{n}-m_{\text{inno}(n-1)}}{n}$$

(47) $$var_{\text{inno}(n)}=\frac{var_{\text{inno}(n-1)}+(\text{inn}o_{n}-m_{\text{inno}(n-1)})(\text{inn}o_{n}-m_{\text{inno}(n)})}{n}$$

(48) $$\sigma_{\text{inno}(n)}=\sqrt{var_{\text{inno}(n)}}$$

where *inno_n_*, *m_inno_*_(_*_n_*_)_, *var_inno_*_(_*_n_*_)_, and *σ_inno_*_(_*_n_*_)_ are the value, mean, variance and standard deviation of the variable *inno* at epoch *n*, respectively.

In some cases, only one or two GPS satellites containing range errors may cause drift in the GPS position and velocity. If the LC scheme is used, these aiding measurements are completely rejected, and the integrated system enters the stand-alone mode with the INS mechanization only. In contrast, if the modified TC scheme is used, only abnormal GPS measurements are eliminated while others continue to be used in data fusion for more accurate estimates. This effect is another advantage of the modified TC scheme compared with the LC and original TC schemes. Traditionally, users can set higher cut-off angles for a GPS receiver to reduce the effect of reflected signals in GPS-degraded regions, even with a low-cost GPS receiver. However, such a procedure may remove normal GPS measurements, thus leaving the low-cost TC integrated INS/GPS system unaided, which consequently degrades the overall position accuracy. However, the proposed algorithms determine the quality of the GPS measurements based on the detection of abnormal pseudo-range errors with the aid of the INS mechanization rather than on the elevations of the GPS satellites.

### 3.4. Software Design

Based on the proposed schemes, the software for processing raw measurements from GPS and the IMU was developed in the C++ programming language in the Microsoft Windows operating system. The major functions of the software include reading raw measurements from GPS receivers and various IMUs, processing the data with the EKF and smoothing in real-time, and post-processing with the LC and TC schemes.

**Figure 9.**
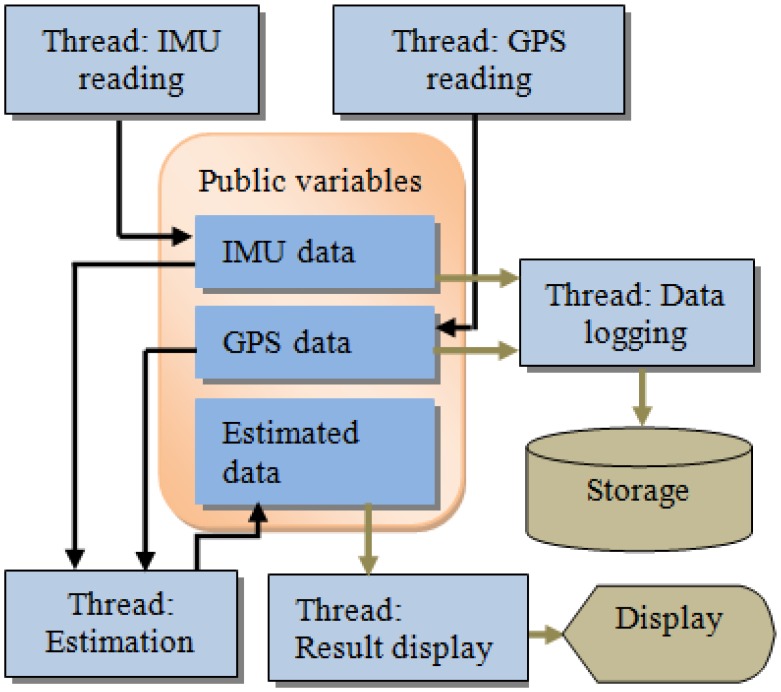
Real-time processing architecture.

For real-time processing, a multi-threaded program is designed to simultaneously implement multiple tasks, including reading data from sensors, real-time processing, logging data for post-processing, and displaying results. Figure 9 shows the architecture of the real-time processing module.

In the data reading engine, IMUs and GPS receivers are supported in the software. Depending on the format of the output data, functions for parsing and decoding the data are designed for a given sensor based on the specified output messages and the protocol specification.

**Figure 10.**
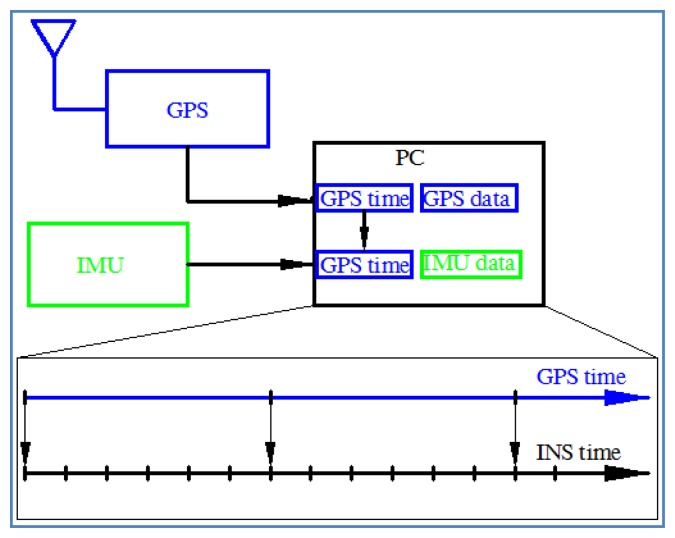
On-line time synchronization.

Time synchronization is very important for an integrated system and for data fusion. In general, the incoming data from different sensors have different time counters, formats, and sampling rates. For integration, time synchronization is necessary. The effects and the methods of time synchronization have been previously investigated [8,14]. In this research, a hardware-based method is implemented. In a real-time system, because incoming data from the IMU and the GPS receiver are handled on the same computational platform (PC) at the same time, the system tags the INS data with the incoming GPS time. Because the sampling rate of the IMU is usually higher than that of the GPS, the time stamp for the incoming inertial data is interpolated based on the IMU sampling rate between two incoming GPS measurements. The times of the INS and GPS data are thus synchronized. Figure 10 shows the on-line time synchronization scheme implemented in this study. The time synchronized error depends on the sampling rate accuracy of IMU and GPS. In extra tests, the time synchronized error between Ublox GPS receiver and MIDG-II IMU (Robotics) is about 1 millisecond and between Propak-V3 GPS receiver (Novatel) with C-MIGITS IMU (BEI) is 0.3 millisecond. These errors are not significant to the overall performance of the system.

## 4. Experiments and Discussion

Field tests with various scenarios were conducted to verify the proposed strategies. In the first test, two integrated systems were set up to evaluate the effectiveness of the proposed method. The reference system comprised a high-end, tactical-grade IMU, a SPAN-LCI (NovAtel), and a dual-frequency, geodetic-grade GNSS receiver, a SPAN-SE (NovAtel). The specifications for the SPAN-LCI IMU are shown in Table 1. The reference data was generated by the reference system with its raw IMU measurements and GPS carrier-phase measurements processed in the differential mode with commercial software, Inertial Explorer (NovAtel), and sensor fusion was performed in the post-processed TC smoothing mode with an odometer. In general, the accuracy of the reference system was less than 10 cm, which is considered sufficient for this study.

**Figure 11.**
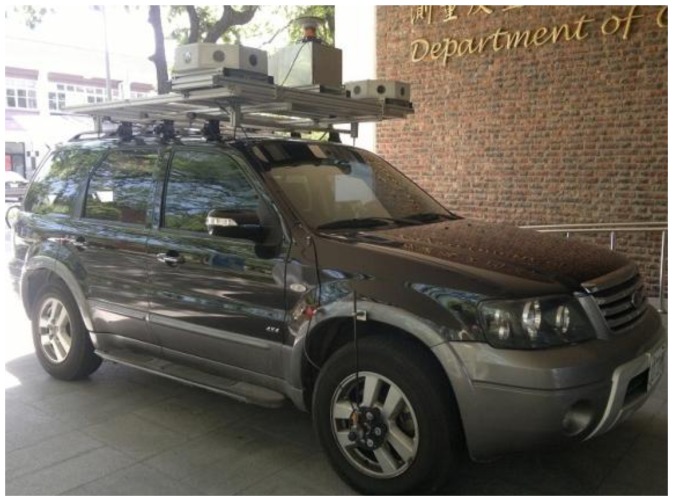
Test van.

The test system comprised a MEMS tactical-grade IMU, the C-MIGITS (BEI), with an integrated dual-frequency GPS receiver with Doppler measurement provided, Propak-V3 (Novatel). The specifications for the test IMU system are shown in Table 2. SPP technique is used to provide GPS measurements for the test. Both two integrated system were mounted on a mobile mapping van (Figure 11) for the field tests.

**Figure 12.**
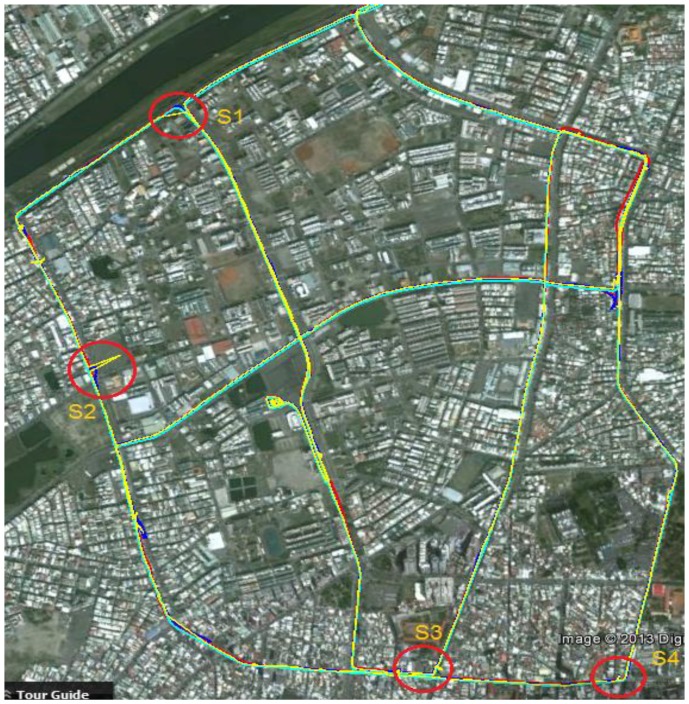
First test trajectory.

**Figure 13.**
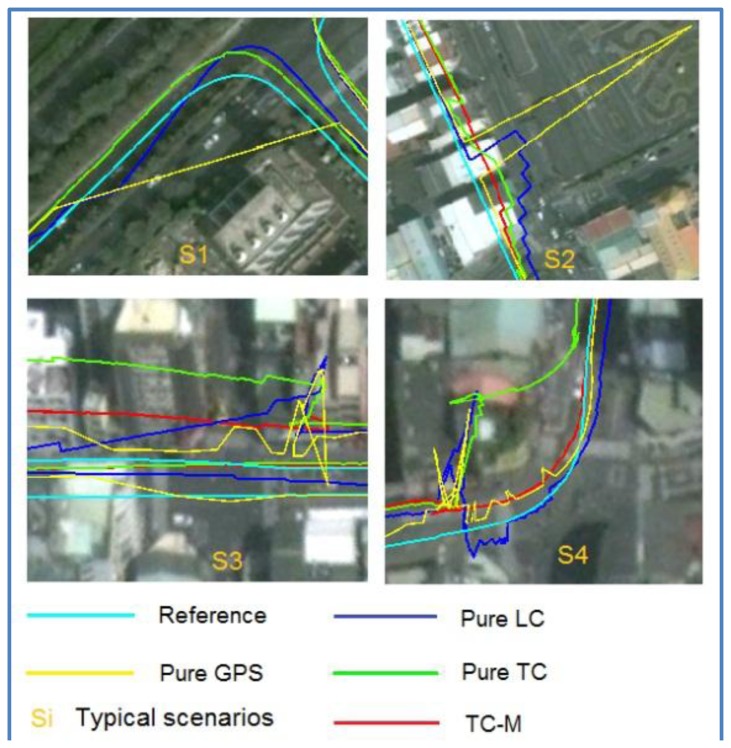
Enlargement of typical scenarios.

**Figure 14.**
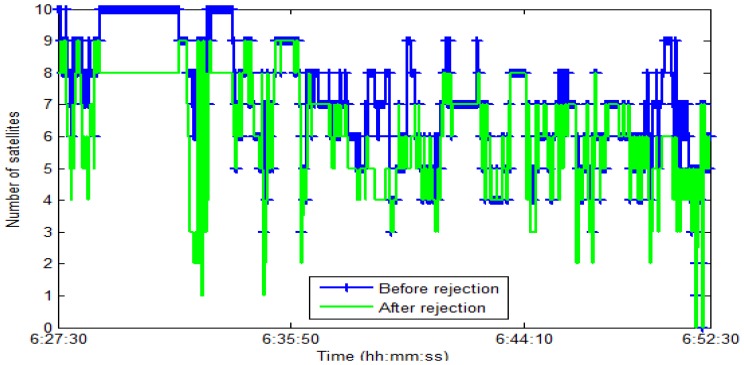
Number of satellites used before and after rejection.

In the first test, the test data sets were collected under various scenarios in urban and suburban areas in Kaohsiung, Taiwan. The test trajectory is shown in Figure 12. Abnormal GPS measurements were included in the test trajectory naturally and by simulation. Figure 13 shows an enlargement of the abnormal GPS measurement scenarios, and Figure 14 shows the number of satellites before and after abnormal GPS measurement rejection.

**Figure 15.**
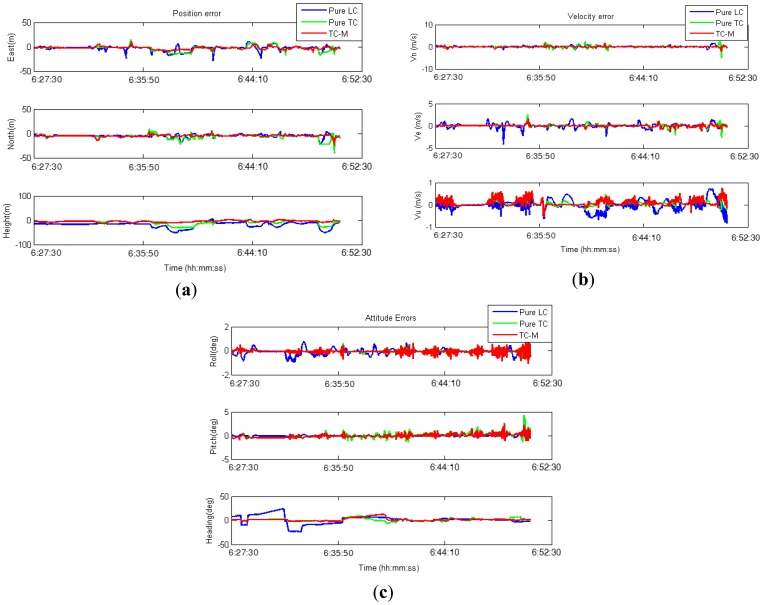
(**a**) Comparison of position error in the first test; (**b**) Comparison of velocity error in the first test; (**c**) Comparison of attitude error in the first test.

For the test scenario, three data fusion strategies, namely, pure LC, pure TC, and modified TC with abnormal GPS measurement rejection (TC-M), were implemented. The estimated position, velocity, and attitude were compared to the reference data for further analysis. Table 3 shows the statistics of root mean square errors (RMSEs), and Figure 15 illustrates the performance of the three algorithms in terms of position, velocity, and attitude errors.

The analysis in the first test indicates that in good GPS signal environments, the performances of the three integrated strategies are comparable. However, in GPS-degraded environments, there are significant differences. As show in Figure 13, GPS measurements are frequently affected by the multipath effect or an insufficient number of satellites to derive navigation solutions in urban canyons. The GPS solutions are biased to large deviations from the reference trajectory. In these scenarios, the navigation solutions of the pure LC and pure TC schemes are strongly influenced because the abnormal GPS measurements are used to update in the EKF. In contrast, the modified TC scheme can detect and ignore abnormal GPS measurements; therefore, its performance is better than that of the pure LC and TC schemes. However, the navigation accuracy of the modified TC scheme slightly decreases in these scenarios because fewer GPS measurements remain to correct the INS mechanization after the bad measurements have been eliminated. Table 3 and Figure 15 show that, overall, the improvement of the pure TC scheme compared with the LC scheme is not significant, with the horizontal accuracy being worse. In GPS-noisy environments, the pure TC scheme is often worse than the pure LC scheme because of the effect of reflected GPS signals. The performance of the proposed modified TC with interference from abnormal GPS measurements is better than that of the LC and pure TC schemes. The improvement in the RMSE of position, velocity, and attitude is approximately 60%.

**Figure 16.**
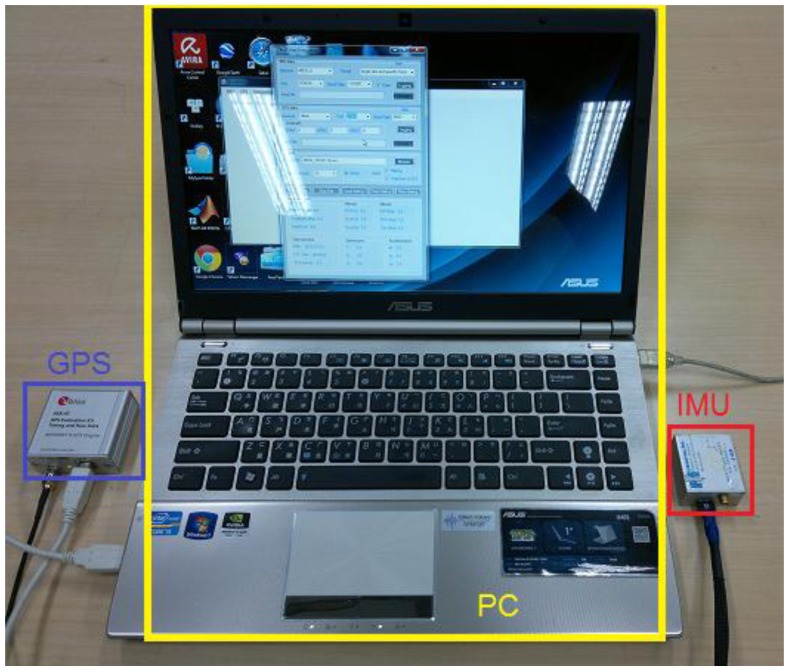
Integrated system in the second test.

**Figure 17.**
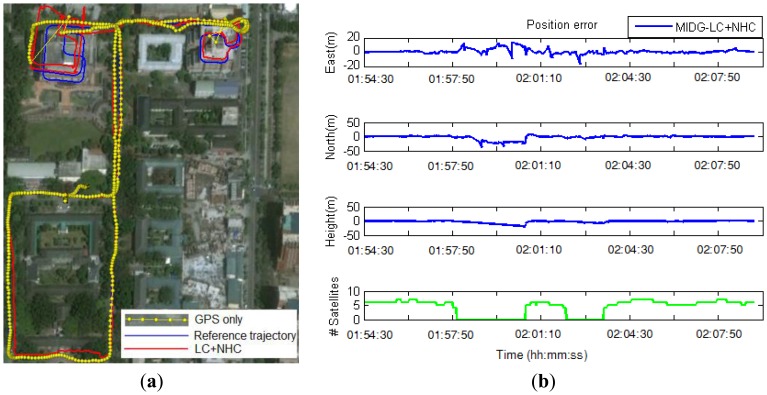
Trajectory (**a**) and position error (**b**) obtained with LC+NHC scheme in the second test.

**Figure 18.**
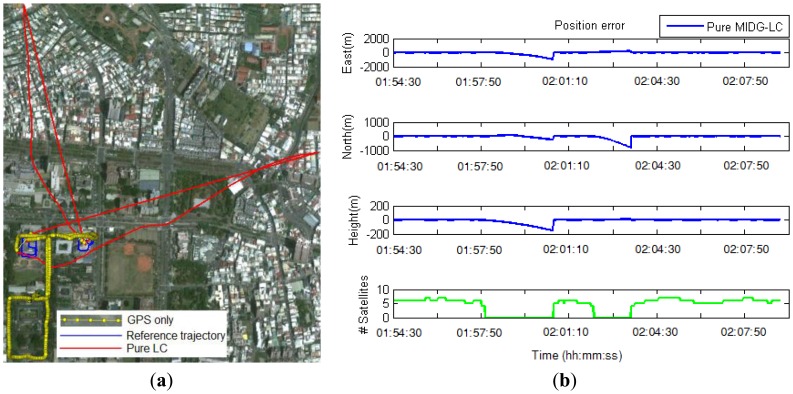
Trajectory (**a**) and position error (**b**) obtained with the pure LC scheme in the second test.

In the second test, a real-time integrated system with an LC + NHC algorithm was analyzed. The test system comprised a low-cost MEMS IMU, the MIDG-II (specifications shown in Table 4), and a single-frequency GPS receiver, the AEK-4T (Ublox) in SPP mode. The two devices were connected to a laptop via the RS232 communication protocol, as shown in Figure 16. The system was installed in the mobile mapping van for the field test. The longest GPS outage, 3 min, was generated by driving the car into an underground parking lot. The test results were compared with those of the reference system for performance analysis. Figure 17 shows the performance of the LC + NHC scheme, Figure 18 shows the performance of the pure LC scheme, and Table 5 shows the navigation accuracy of two integration strategies.

The preliminary results from the second test indicate that under open-sky conditions, the position accuracy of the proposed system mainly depends on the accuracy of GPS. The positional accuracy of the integrated system in that case is about 1 to 2 m. In GPS-rejected environments, the navigation solutions of the system completely rely on the output of the INS and aiding sources. With the given MEMS IMU in the second test, in the pure LC mode, the position error grows quickly over time if no GPS updates are available. The position error is approximately 25 m after a 15-s GPS outage. In a long-duration GPS outage (3 min, in this test), the position error drifts to a very large value because of the divergence in the estimator. In contrast, with the augmentation of NHC, for a 3-min GPS outage, the maximum position error is approximately 20 m and the overall position accuracy is approximately 10 m.

Generally speaking, the performance of the integrated system depends on the performance of the IMU and the GPS receiver, the operating environment, and the data fusion strategy. In open-sky conditions, the position accuracy mainly depends on the accuracy of the GPS. Therefore, improving the positioning technique and eliminating the GPS systematic errors are the keys to improving the overall navigation accuracy of the system. In GPS-degraded and -denied environments, the performance of an IMU with a data fusion strategy generally affects the final solutions. Because the goal of this research is to improve the performance of the system using a low-cost MEMS IMU, the data fusion strategy is most critical. As shown in the first test, the overall position accuracy improved by approximately 60% in a GPS-degraded environment by improving the estimation strategy. The position accuracy can reach 10 m with a 3-min GPS outage, as was shown in the second test using a low-cost MEMS IMU with the aid of the NHC in GPS-denied environments.

## 5. Conclusions

This study developed a real-time integrated Inertial Navigation System (INS)/Global Positioning System (GPS) navigation system for land vehicle applications. Issues related to GPS uncertainty and the systematic errors of an Inertial Measurement Unit (IMU) were investigated. A modified tightly coupled (TC) integration scheme was used to reject abnormal GPS measurements. Two Micro-Electro-Mechanical Systems (MEMS) IMUs were used for testing under various scenarios.

The preliminary results showed the effectiveness of the proposed strategies. Overall, for the INS/GPS integration mode, the improvement of the modified TC scheme over the standard loosely coupled (LC) and pure TC schemes is approximately 60% in terms of position uncertainty. The main advantage of the proposed system is that it can detect and eliminate abnormal GPS measurements, mainly caused by the multipath effect, thus improving the overall performance of the integrated system. In cases of long-duration GPS signal outages, with the aid of the Non-Holonomic Constraint (NHC), the integrated system using a low-cost MEMS IMU can provide reliable solutions for navigation applications.

## Figures and Tables

**Table 1.** The specifications for the reference system.

**Physical Characteristics**	**IMU Performance**	
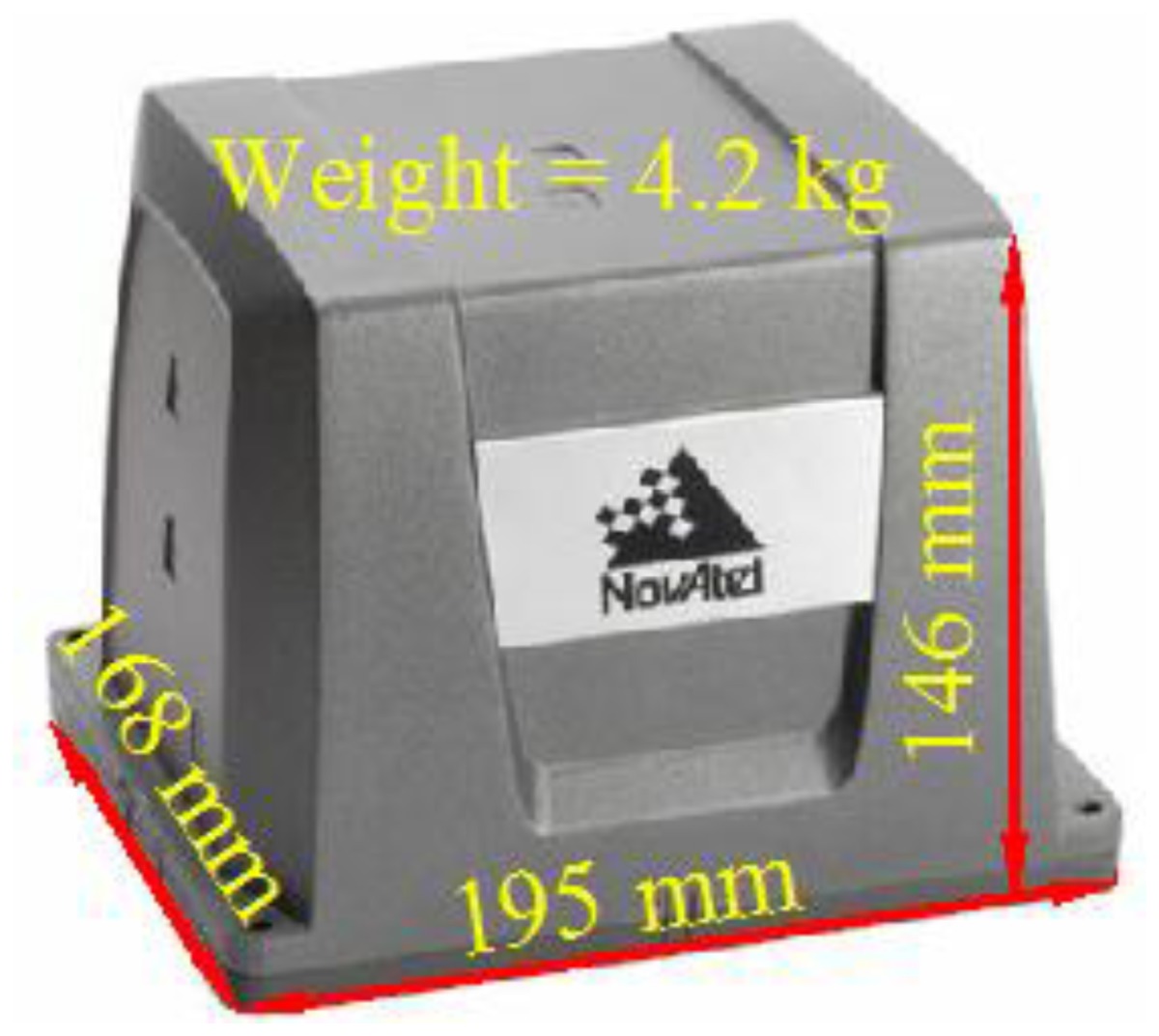	Output rate (Hz)	200
	In run Gyro bias (degrees/h)	0.1
	Gyro scale factor (ppm)	100
	Accelerometer bias (milli-g)	1.0
	Accelerometer scale factor (ppm)	250

**Table 2.** The specifications for the test system.

**Physical Characteristics**	**IMU Performance**	
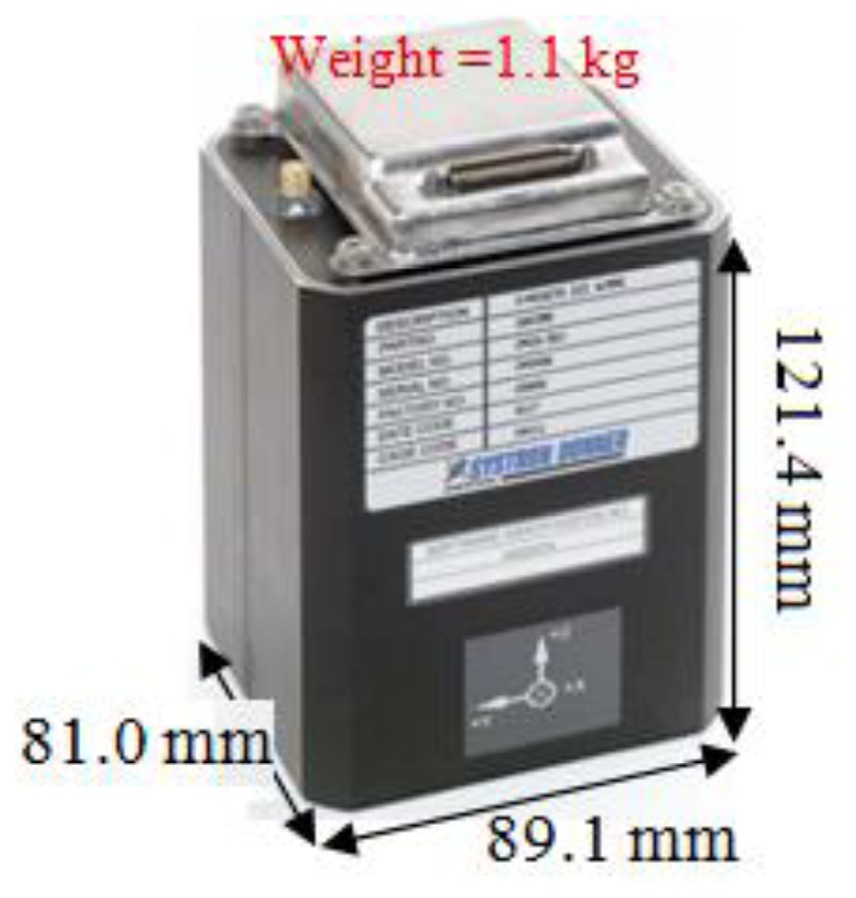	Output rate (Hz)	100
	In Run Gyro bias (degrees/h)	3∼5
	Gyro scale factor (ppm)	350
	Accelerometer bias (milli-g)	4
	Accelerometer scale factor (ppm)	350

**Table 3.** Comparison of RMSE, LC and TC schemes.

**RMSE**	**LC**	**Pure TC**	**TC-M**
**East (m)**	6.0	6.0	3.9
**North (m)**	5.9	7.9	5.4
**Up (m)**	22.3	12.6	6.3
**3D (m)**	23.8	16.1	9.2
**Improvement compared to LC (%)**		33	61

**Vx (m/s)**	0.35	0.49	0.28
**Vy (m/s)**	0.54	0.32	0.22
**Vz (m/s)**	0.22	0.15	0.16
**3D (m/s)**	0.68	0.60	0.39
**Improvement compared to LC (%)**		11	48

**Roll (°)**	0.26	0.13	0.12
**Pitch (°)**	0.15	0.47	0.35
**Heading (°)**	9.02	3.64	3.05
**3D (°)**	9.02	3.67	3.07
**Improvement compared to LC (%)**		59	66

**Table 4.** MIDG-II specifications.

**Physical Characteristics**	**IMU Performance**	
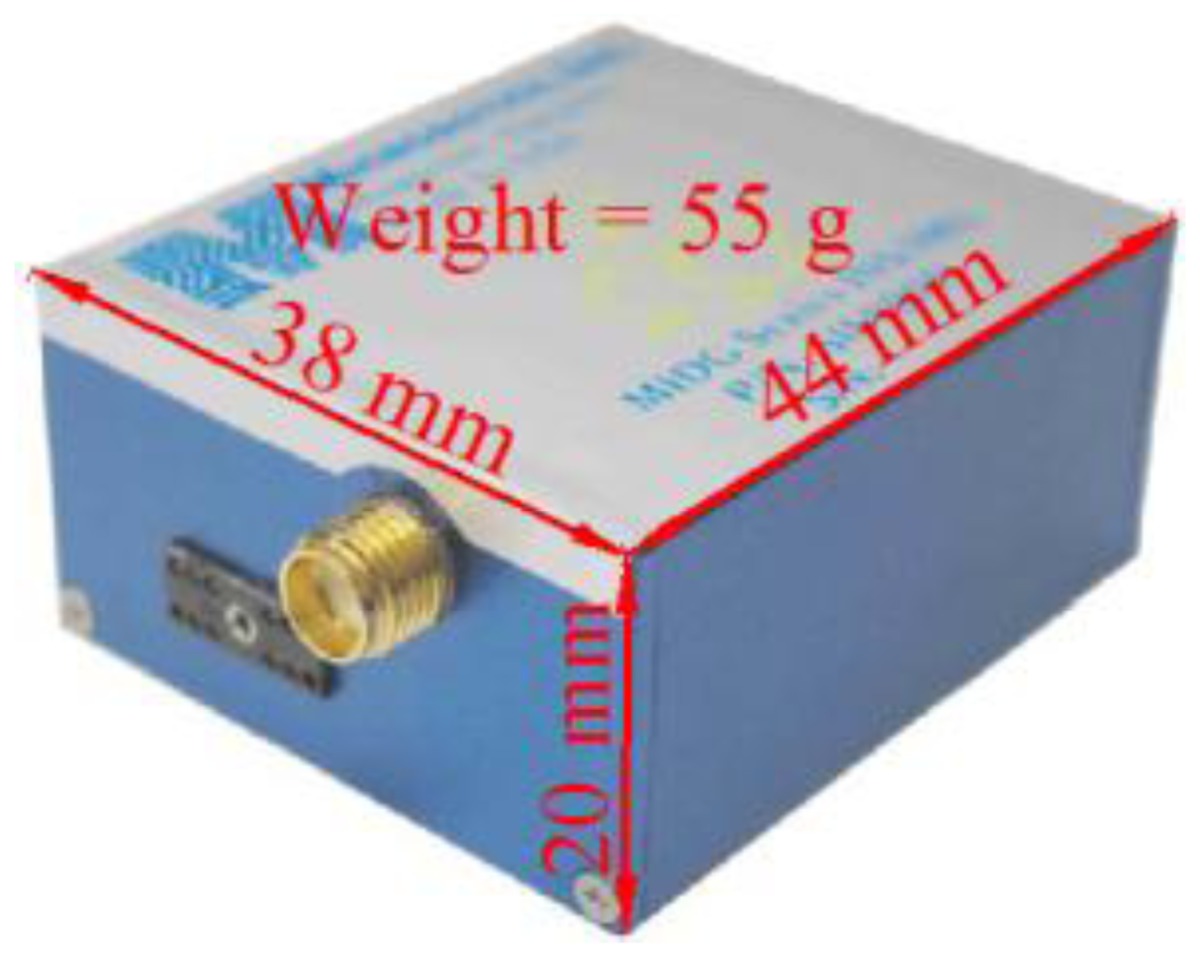	Output rate (Hz)	50
	Gyro bias (degrees/h)	47
	Gyro scale factor (ppm)	5,000
	Accelerometer bias (milli-g)	6.0
	Accelerometer scale factor (ppm)	19,700

**Table 5.** Performance of the system in the second test.

**RMSE**	**Pure LC**	**LC** + **NHC**	**Improvement of LC** + **NHC over LC (%)**
East (m)	192.1	3.5	98
North (m)	140.6	7.9	94
Up (m)	34.1	5.4	84
3D (m)	240.5	10.2	96
